# Editorial: Translational brain-computer interfaces: From research labs to the market and back

**DOI:** 10.3389/fnhum.2023.1152466

**Published:** 2023-02-13

**Authors:** Davide Valeriani, Hubert Cecotti, Antonia Thelen, Christian Herff

**Affiliations:** ^1^Google LLC, Mountain View, CA, United States; ^2^Department of Computer Science, California State University, Fresno, CA, United States; ^3^eeimagine Medical Imaging Solutions GmbH, Berlin, Germany; ^4^Department of Neurosurgery, Maastricht University, Maastricht, Netherlands

**Keywords:** brain-computer interface, neurotechnology and brain-machine interface, EEG, fNIRS, neuromarketing, BCI, human-computer interaction

Neurotechnologies combine neuroscience and engineering to build systems for studying, repairing, and augmenting human performance. These include brain-computer interfaces (BCIs)—typically used as assistive devices for communication and rehabilitation for disabled people and, more recently, as human enhancement devices—neural prosthetics, optogenetics, digital medicine, continuous remote monitoring, drugs, and other pharmaceutical interventions.

This Research Topic provides a collection of nine novel contributions on recent bidirectional interactions between academic labs and industry partners to showcase translational work in BCIs for clinical, business-to-business, and consumer markets that push neurotechnologies outside of the lab.

In the context of neuromarketing, Zeng et al. showed that whether users like or dislike a pair of sport shoes could be understood by looking at their electroencephalography (EEG) activity. Amongst all feature extraction methods explored, they found that differential entropy features extracted from signals recorded from the occipital region provided the highest classification accuracy in detecting shoes that the user liked. The importance of the occipital cortex in visual processing and emotion arousal is well-known in neuroscience literature (Lang et al., 1998). An auditory task may instead have involved the temporal region more heavily than the occipital one. The authors demonstrated that the frontal region also played an important role in boosting classification accuracy, as the decision-making process of the user occurs in this area (Volz et al., 2006). This study makes a first step toward using passive BCI systems as tools for gathering user feedback on consumer products, without specifically asking to rate them through traditional reviews. Future studies need to explore if similar results could be obtained using consumer EEG devices with dry electrodes, instead of research grade wet EEG systems as used in the study.

The process of porting BCIs from research labs to the market encompasses the development of easy to use, reliable, and cost effective sensors that could replace the usually bulky, expensive, and difficult to setup research-grade EEG systems. An attempt to respond to this need has been made by Sciaraffa et al., who have designed and tested a BCI system composed of water-based electrodes and a lightweight headset. They found this system to provide comparable results to BCIs based on research-grade EEG systems and gel-based electrodes across a variety of passive tasks and environments, including multitasking, psychomotor vigilance task, and car driving simulation. The usage of water electrodes increased ease of use without lowering performance, and the lightweight headset made the BCI comfortable enough for many real-life applications. Another great challenge in BCI translation is reducing the training time, i.e., the calibration session, enabling users to benefit from the BCI as soon as possible. A key technique in this domain is transfer learning, namely reusing some of the information gathered from a previous training session to train a new model for a different task or user. Bleuzé et al. exploited Riemannian geometry to develop a new transfer learning method for BCIs that identifies who is the most similar subject to the user at hand in the previous training session, and uses these parameters for the new model. They extensively tested this approach on 18 BCI databases, finding a significant improvement on BCI performance over standard training-test pipelines, including those using Riemannian geometry.

A typical BCI application out of the labs is their use in entertainment. A study in this Research Topic explored the interaction between brain and heart through the heart-evoked potential in gamers and non-gamers playing a video game (Khoshnoud et al.). They found that flow—the state of complete absorption in an activity—can be tracked through EEG sensors and does not differ between gamers and non-gamers, as long as the user is able to set the level of difficulty of the video game. Another study tracked the process of musical transposing (i.e., converting notes across musical keys) in musicians and found it significantly differs from math calculations, although both tasks involve the usage of working memory (Lu et al.). Another novel study explored the usage of BCIs for story generation through increases of frontal alpha asymmetry (Krogmeier et al.). Although only 37% of the participants were able to successfully modulate their brain activity during story creation, this novel paradigm further reveals the importance of understanding BCI literacy and what factors make users able to control a novel BCI successfully.

As BCIs were born as human-computer interaction technologies, another great area of impact of BCIs out of the lab is helping patients with serious neurological disorders regain their independence. A pilot study by Riccio et al. explored the use of BCIs in patients with multiple sclerosis, as a possible integration with other assistive devices designed for these patients. The majority of the patients were able to control an assistive device software with their brain activity, paving the way to the integration of BCIs into the daily life of multiple sclerosis patients. While Riccio et al. used an established paradigm of BCI based on the detection of event-related potentials (the P300 speller), Klee et al. explored the usage of occipitoparietal alpha activity in a speller based on rapid serial visual presentation (RSVP). This novel paradigm for communication performed better than random, but well-below more established paradigms for spellers. Future investigations should test the possibility of combining these paradigms into a hybrid approach, which may lead to superior performance than other paradigms. Combining different types of assistive devices seem to be the next frontier for BCIs, as presented by Eagleman and Perrotta in their perspective on the future of sensory substitution, addition and expansion *via* haptic devices, which indeed represent very promising technologies for human rehabilitation and augmentation.

## Author contributions

DV drafted the manuscript. All authors provided feedback and contributed to the Research Topic. All authors contributed to the article and approved the submitted version.
